# Reason and less

**DOI:** 10.3389/fpsyg.2014.00901

**Published:** 2014-08-20

**Authors:** Vinod Goel

**Affiliations:** ^1^Department of Psychology, York University, TorontoON, Canada; ^2^Istituto di Ricovero e Cura a Carattere Scientifico-Fondazione Ospedale San CamilloVenice, Italy

**Keywords:** rationality, reasoning, Decision Making, evolutionary psychology, instincts, biases

## Abstract

We consider ourselves to be rational beings. We feel that our choices, decisions, and actions are selected from a flexible array of possibilities, based upon reasons. When we vote for a political candidate, it is because they share our views on certain critical issues. When we hire an individual for a job, it is because they are the best qualified. However, if this is true, why does an analysis of the direction of shift in the timbre of the voice of political candidates during an exchange or debate, predict the winner of American presidential elections? Why is it that while only 3% of the American population consists of white men over 6′4″ tall, 30% of the CEOs of Fortune 500 companies are white men over 6′4″ tall? These are examples of “instinctual biases” affecting or modulating rational thought processes. I argue that existing theories of reasoning cannot substantively accommodate these ubiquitous, real-world phenomena. Failure to recognize and incorporate these types of phenomena into the study of human reasoning results in a distorted understanding of rationality. The goal of this article is to draw attention to these types of phenomena and propose an “adulterated rationality” account of reasoning as a first step in trying to explain them.

Nature, Mr. Allnutt, is what we were put on this world to rise above.

–Katharine Hepburn to Humphrey Bogart in *The African Queen*

## INTRODUCTION AND BACKGROUND

The conception of man as a rational, thinking being, permeates Western thought from (at least) Aristotle to present times. Our behavior is explained by postulating beliefs and desires, and a principle of “rationality,” that guides our pursuit of the latter in the context of the former. My use of the term “rationality” is derived from the philosophical literature, meaning roughly, deliberate reason. (It does not imply a commitment to any normative standard, as is the case in the psychology literature.) On this account rationality is goal directed behavior. It is simply a means to an end and is ascribed to individual agents. It is a deliberate choice or action that moves an organism closer to its goal in a manner consistent with its knowledge and beliefs. A rational choice is not simply a selection, it is a selection for a reason (Bermudez, 2002). Perhaps the most significant feature of a rational system is the existence of a “gap” between the stimulus and the response (Cassirer, 1944). The stimulus or antecedent condition is never causally sufficient to determine any specific choice or action. I will use the term “reason-based” to refer to this general notion of rationality. Reason-based choice is often contrasted with instinctual or tropistic behavior, where there is no such gap and the antecedent conditions are causally sufficient for a course of action (Bermudez, 2002).

Consider the following example: when a young male lion chases away an older male and takes over a pride, he proceeds to kill any cubs the females may be nursing. How should we explain this behavior? Does the lion sit down and reason thus: “these cubs do not perpetuate my genes. They will require the expenditure of considerable resources to feed and defend. Providing these resources to perpetuate someone else’s genes does not make evolutionary sense. However, if I kill these cubs (which I surely can, without harm to myself), the females will stop lactating and come into heat again. I can then impregnate them with my sperm and then they will bear my offspring. Then the resources of the pride can be used to propagate my genes rather than someone else’s. Therefore, it is reasonable to kill these cubs.”

If the lion did deliberate in this way, we would be justified in saying his behavior, however, cruel, was rational. If he reasoned thus, and did not kill the cubs, his behavior would be irrational. But most of us do not believe that the lion has the ability to reason in this manner. Most of us do not believe that the lion chooses actions from a vast array of possible alternatives *for reasons*. His behavior is compelled, in the context of particular environmental and developmental factors. Therefore, applying the label of “rationality” (given the above definition) to explain this behavior is both unnecessary and incorrect. The lion’s behavior is certainly adaptive, but it is not rational or reason-based. It is explained by appeals to instinctual or tropistic mechanisms (such as parental investment parasitization prevention) that are triggered by causal interactions between the maturation of specific internal structures and environmental cues. Once the mechanisms are triggered, they lead to a particular course of action.

Now consider the case of a man who partners with a woman with young children from a previous partner. The man does not typically kill the children, though it may be, arguably, adaptive to do so. Why not? Presumably because he’s making a conscious choice from a wide range of possibilities. He is not driven to an inevitable action. He could choose not to get involved in this relationship and find a woman without children. Perhaps he loves the children. Perhaps he finds the woman so desirable that the opportunity to have his own children with her is worth the price of expending some resources to raise her children from a previous partner. Perhaps his overtures to women without children have been unsuccessful. Whatever the reason, he is making a conscious, rational/reason-based choice.

However, if this appeal to reason is adequate to explain the behavior of the man, why is it the case that instances of child abuse/mistreatment are much higher in the case of stepfathers (and stepmothers) than biological fathers and mothers (Daly and Wilson, 2005)? Why is it the case that, despite our convictions that we vote for political candidates because they share our views on certain critical issues, that a simple analysis of the direction of shift in the timbre of the voice of candidates during an exchange or debate, predicts the winner of American presidential elections (Gregory and Gallagher, 2002)? Why is it the case that, despite our beliefs that we hire individuals for jobs because they are the best qualified, 30% of the CEOs of Fortune 500 companies are white men over 6′4″ tall, even though they represent only 3% of the American population (Rauch, 1995)?

These are all examples of what we might call “instinctual biases” affecting our reasoning and decision-making processes (Buss, 2005). They are genuine, ubiquitous phenomena. But they are not phenomena typically studied by cognitive psychologists interested in human reasoning. Our current research programs either (1) ignore these types of “biases” (Rips, 1994; Johnson-Laird, 2006); (2) assume that they are cut from the same cloth as the conceptual biases in the Linda problem (see below) and can be explained in the same fashion (Evans and Over, 1996; Stanovich, 2004); (3) focus on instinctual biases, but assume that is all there is to human reasoning (Cosmides and Tooby, 1994b; Duchaine et al., 2001) or (4) consider them to be social biases built on top of the cognitive engine and as such do not influence the operation of that engine (Berry, 2007).

I want to suggest that ignoring these phenomena excludes much of what is interesting about human reasoning from our research programs, and may, in fact, result in distorted theories of human reasoning based upon incomplete data sets. Furthermore, if evolutionary psychologists are correct, the effect of biological markers such as dominance cues, facial attractiveness cues, waist to hip ratios (in women), shoulder to waist ratios (in men), etc. are not socially construed phenomena, but apply universally (Buss, 2005). The two theories of reasoning best situated to account for these phenomena are massive modularity theory (Cosmides and Tooby, 1994b) and dual mechanism theories (Sloman, 1996; Evans, 2003; Stanovich, 2004). I argue that neither of these accounts can adequately accommodate the phenomena and propose a banal adulterated rationality account.

## CONCEPTUAL SPACE OF THEORIES OF HUMAN REASONING

### INFORMATION PROCESSING THEORY

Classical information processing theory holds that the cognitive system is a general purpose information processing system, perhaps with some specialized modules, for language and perceptual processes (Fodor, 1983; Pylyshyn, 1984; Newell, 1990). In the context of this framework there are several accounts of reasoning such as mental model theory (Johnson-Laird, 2006) and mental logic theory (Braine, 1978; Rips, 1994). While there are significant differences between these two theories, in terms of the nature of the representations and computations employed during logical reasoning, both postulate a mechanism that operates within the rules of formal logic. These theories generally do not try to explain the phenomenon of “instinctual biases” of interest here.

### MASSIVE MODULARITY

There is a research program that explicitly sets out to account for instinctual biases. Indeed, 20 years ago Cosmides and Tooby (a) exhorted cognitive scientists not to be blind to the effect of instincts (“instinct blindness”). With respect to reasoning, they have worked largely with the Wason card selection task (Cosmides, 1989; Fiddick et al., 2000). The Wason card selection task is a disguised form of conditional inference. Four cards, corresponding to the four forms of the conditional (modus ponens, modus tollens, denying the antecedent, and affirming the consequent) are placed in front of the subject, along with the conditional rule. The basic result is that switching from a rule with arbitrary content (e.g., “if the letter on one side of the card is a vowel, then the number on the other side must be even”) to a rule that embodies the structure of some social contract (e.g., “if someone is drinking beer, then they must be over 18 years of age”), increases performance accuracy from the order of 6% to the order of 80% (Wason and Shapiro, 1971; Cox and Griggs, 1982). The explanation is that this dramatic shift in performance is the result of the triggering of a “cheater detection” module.

On the massive modularity account the mind is not a general purpose information processing system, but rather consists of 1000s of special-purpose modules selected for the adaptive advantage they conferred upon our Pleistocene ancestors in solving problems specific to their environment, such as selecting mates, leaders, and detecting cheaters (Pinker, 1997; Duchaine et al., 2001). The modules are causally triggered by specific environmental cues. In previous times we would have called these modules instincts. Today we might liken them to the apps on our smartphones (Kurzban, 2012). Like apps they work relatively independently, though they may have access to information generated by other specific apps. For example, the app that I use to monitor my walks has access to information from the GPS and the system clock. It does not have (nor requires) access to the output of the apps that I use to listen to audiobooks or track flight arrivals. One can of course imagine a greater degree of interdependence and interaction among modules, but the main point is that there is no general-purpose reasoning system that controls the selection and triggering of individual modules. The selection and triggering are determined by direct causal links to specific environmental cues. On a strong version of the account, it is claimed that our notion of rationality (or general purpose reasoning) is illusory. What we regard as “general purpose reasoning” is just the functioning of numerous instinctual modules (Cosmides and Tooby, 1994b).

Several authors offer compelling critiques of the massive modularity account (Fodor, 2001; Over, 2002). I believe its greatest strength is that it offers a potential solution to the intractable problem of induction (or the frame problem) that plagues cognitive psychology, albeit at the price of a tight causal coupling between specific environmental cues and triggering of specific modules. Its greatest weakness is that it cannot explain how we can send a man to the moon and predict and investigate the existence of the Higgs boson (because presumably nothing in the Pleistocene environment of our ancestors would have selected for these abilities).

But for present purposes, I will limit my concerns about massive modularity to its ability to explain the specific examples of reasoning/decision-making with which I began. It is not clear that the above examples can be explained *just* in terms of activation of specific instinctive modules. One reason to doubt the ability of the massive modularity model to explain the phenomenon of interest is to note the differences in the response patterns, and the ability of participants to reflect upon and justify their responses, in the case of our examples and the Wason card selection task.

In the case of the Wason card selection task, a shift in content to a rule breaking scenario results in a shift in accuracy approaching ceiling level, and one can plausibly argue that the shift in content triggers something like a cheater detection module. However, this does not seem to be the case for the examples in the introduction. For example, while instances of child abuse by stepparents are significantly greater than for biological parents, they do not approach 80% (Daly and Wilson, 2005). We will not typically vote for a leader who intends to adversely affect our lives, despite the presence of dominance cues. In hiring a doctor we would not typically choose a tall, handsome, athletic man without a medical degree over a short, hunchbacked, pudgy man with a medical degree. Therefore these phenomena cannot be explained just in terms of an appeal to instinctual modules (as they can in the case of the lion, and perhaps even the Wason card selection task). Such phenomena call for *a blended response* between instincts/modules and some general purpose reasoning system.

There is also a discrepancy between the response/behavior and the reason/explanation offered for the behavior. In the case of the Wason card selection task, participants can typically articulate why they chose particular cards (in the familiar content or cheater detection version). In the case where we are evaluating two potential employees (or grad students) with similar views and qualifications, if one exhibits high attractiveness cues, while the other does not, we will often choose the attractive individual, but when questioned, our explanation will not implicate these cues. It will be in terms of the qualifications of the candidates, even though there may be no material differences in these factors (Dipboye et al., 1975; Langlois et al., 2000; Hosoda et al., 2003). We will not be consciously aware of the effect of the attractiveness cues on our reasoning/decision-making behavior. This again suggests that there are at least two processes at work here, a conscious general-purpose reasoning system that evaluates the qualifications of the two candidates, and unconscious instinctual biases that modulate the operation of the former system.

It may be tempting to draw parallels between our inability to report on the causal efficaciousness of instinctual biases and the confabulation that split brain patients engage in when the verbal left hemisphere is unaware of the choices made by the right hemisphere. While there are some similarities, the dissimilarities may be greater. Consider the following famous experiment (Gazzaniga, 1998): a split brain patient was presented with a picture of a winter scene projected to the right hemisphere (left visual field) and a picture of a chicken claw projected to the left hemisphere (right visual field). The patient must then select two related pictures, one picture with each hand, from an array of other pictures. The patient’s left hand points to a shovel (because the right-hemisphere, controlling that hand has seen a snow-covered winter scene) and the right-hand points to a chicken (because the left hemisphere, controlling that hand, has seen the chicken claw). When the patient is asked to explain why his left hand (guided by the right hemisphere) is pointing to the shovel, the left hemisphere (dominant for language) has no access to the information about the winter scene seen by the right hemisphere. But instead of responding “I don’t know” the patient responds by noting that the shovel is required to clean the chicken coop.

The similarity lies in the fact that in both cases, the verbal explanation for the behavior cannot causally account for the behavior. The dissimilarity is that the explanation offered by the left hemisphere of the split brain patient is a complete *post hoc* confabulation. It simply is not relevant to explaining the behavior. In the case of instinctual biases, we are not “confabulating” in the same sense because the conscious explanation that we offer (e.g., “this applicant has a degree from University of Waterloo”) is usually causally relevant. It cannot explain the complete pattern of the data, but it may be a relevant part of the causal story.

### DUAL MECHANISM THEORIES

There is a research program that acknowledges the necessity of a general-purpose reasoning system and also explicitly sets out to account for various reasoning biases. These dual systems accounts of reasoning contrast heuristic/intuitive (System 1) processes with formal (System 2) processes (Sloman, 1996; Evans, 2003; Stanovich, 2004). This is becoming a widely accepted distinction and seems to have an underlying neuropsychological basis (Goel and Dolan, 2003; Goel, 2007). The critical feature of this paradigm is that while there is a logical/formal response to the task, in some conditions it is inhibited and bypassed by subjects’ *background knowledge and beliefs*. An example is provided by the famous Linda Problem (Tversky and Kahneman, 1983):

Linda is 31 years old, single, outspoken, and very bright. She majored in philosophy. As a student, she was deeply concerned with issues of discrimination and social justice, and also participated in anti-nuclear demonstrations.

Which statement is most likely?

(a) Linda is a bank teller and active in the feminist movement

(b) Linda is a bank teller

The effect is that many intelligent individuals will choose the conjunction (a) as more likely than one of the conjuncts (b). Their rationale is that the conjunct (b) by itself does not seem sufficient for someone with Linda’s background. The conjunction (a) in addition contains a conjunct that seems more appropriate given the background description of Linda. The usual explanation for the “irrational” response is that overall (a) is more “representative” of Linda than (b) (even though a conjunction cannot be more likely than either conjunct)^1^. This has led to a distinction between formal processes and heuristic/intuitive processes (Evans, 2003; Stanovich, 2004).

This is a genuine, important psychological phenomenon. Some dual mechanism theorists have argued that these heuristic responses represent primitive, low-level instinctual biases that we share with pigeons and rats (Evans and Over, 1996). This is simply a mistake. The bias exhibited in the Linda problem is a very high level, conceptual bias based upon language and our knowledge of the world. Note that while heuristic responses may be considered irrational, in sense of violating normative logic, [though this is a moot point (Politzer and Noveck, 1991; Gigerenzer, 2007; Goel, 2008)], both responses are clearly reason-based, as I am using the term here. There are sensible psychosocial expectancy reasons for why subjects choose the so-called “irrational” response. If the logical inconsistency of their response is pointed out to subjects, they can quickly give the logically correct response and offer justifications for the initial heuristic response (Sloman, 1996). My conjecture is that instinctual biases are drawn from a very different well than the conceptual biases exhibited in the Linda problem. If this is the case, there is no reason to believe that the theory can account for the types of reasoning phenomenon of interest here.

One response to this objection is to note that System 1 is a heterogeneous collection of everything from reflex arcs to conceptual biases (Stanovich, 2004). In this case, the instinctual biases I am trying to bring attention to would fall into System 1 (as would many reason-based processes). Even though it has been argued elsewhere (Goel, 2008), that the differences in the underlying causal mechanisms of such a heterogeneous collection of System 1 processes makes the category uninteresting for theory building, the distinction is considered useful because System 1 processes are said to share behavioral similarities in outputs in terms of speed and automaticity of responses (Stanovich, 2004). Here I want to suggest that the behavioral patterns are very different in the case of conceptual biases and instinctual biases. The argument here is similar to that offered above for the massive modularity account.

The whole point of dual mechanism accounts is that the processing goes through one of the two systems. The responses are *either* “rational” *or* “heuristic.” This model works well for the type of phenomena the theory was intended to explain, such as content effects in syllogisms (Evans, 2003) and the Linda problem (Tversky and Kahneman, 1983). In each of these cases the conceptual biases result in a dramatic shift in subject responses, so perhaps one can argue that the bias results in the queuing of a different system. Furthermore, individuals are consciously aware of and can articulate the reasons for the “non-rational” response (Sloman, 1996).

In the case of the instinctual biases of interest here, there is neither a dramatic shift in behavior such that 90% of participants are responding in one way or the other, nor an awareness of the reasons for the behavioral shift. Both of these points have been illustrated with examples in the above discussion of massive modularity. As in the case of massive modularity, the dual mechanism accounts work best when there is a dramatic shift in performance (as in the Linda Problem) but will require some sort of modulation/interaction account where the response shift is graded and less pronounced, and subjects are unable to fully articulate causally efficacious reasons for their response/choice.

### ADULTERATED RATIONALITY ACCOUNT

I think the key missing feature in the above accounts of human reasoning is the recognition of the modulation of rational choice by instinctual biases. Any theory that is going to do justice to human reasoning must acknowledge both a rational system and a host of instinctual systems or biases. It must also acknowledge that these systems interact, to varying degrees, in human reasoning and decision-making. Human choices cannot be explained by postulating a single type of system, whether it be instinctual modules or a general-purpose reasoning system.

I am proposing a banal model whereby the rational engine has evolved on top of instinctual/tropistic mechanisms. The nature of these instinctive mechanisms can perhaps be understood along the lines of the “automatic appetitive impulsive processes” postulated in the addiction literature (Gladwin et al., 2011; Wiers et al., 2013). The instinctual biases of interest here need not be “appetitive” processes, but they are automatic, impulsive, non-cognitive processes that manifest individual differences, and modulate and in turn are modulated by, top-down (reason-based) executive control processes. Thus functioning of the rational engine is modulated or adulterated by these processes to varying degrees, depending on the nature of the tasks, and individual differences. For example, the rational engine would be more affected by instinctual biases in the case of mate selection than in calculating the launch trajectory of a satellite to orbit Mars. I view this process of modulation or adulteration as one of bending and warping the architecture of the reason-based system such that certain possibilities are facilitated, hindered, or even blocked. I propose to call this the “adulterated rationality” account of reasoning.

The system is set up in such a way that the unconscious bottom–up instinctual biases or modules are triggered by task specific cues in the environment (along the lines postulated by the massive modularity account), however, rather than being the sole determinants of behavior, these biases pass through a conscious top-down reason-based system, resulting in a response that is a blended product of the two systems. Individual differences in the strength of specific bottom-up, non-cognitive, instinctual biases, and the strength of top-down cognitive, reason-based processes and strategies, along with the nature of the reasoning task, will affect the ratio of the mixture.

For example, consider the discriminative parental solicitude effect with which we began. Parental investment is a valuable resource that can be parasitized by non-relatives (Daly and Wilson, 1994). It is suggested that we all have mandatory, automatic, innate mechanisms for countering parental investment parasitism (Daly and Wilson, 1994). These mechanisms must be suppressed in the case of stepfathers (and stepmothers) where they make the conscious decision to accept a mate with children from another partner. The majority of stepfathers and stepmothers are able to bond (to some extent) with their new mate’s existing offspring, but there is considerable individual variability. The standard explanation for failure would implicate top down inhibition processes (i.e., “they didn’t try hard enough”). But an equally likely possibility is variability in the strength of the mandatory impulsive, non-cognitive, bottom-up processes. If these instinctual systems are exceptionally strong in certain individuals, then an equivalent exertion of top-down processes will not result in the same effect. This raises some interesting psychological, biological, ethical, and legal issues.

## CONCLUSION

My goal here has been to draw attention to an ubiquitous, but neglected phenomenon which affects our rational behavior: the modulation of conscious rational choice by unconscious instinctual biases. Much of the study of human rationality within cognitive psychology has focused on logical form. It is time to look beyond logical form. Recent studies directed at the role of emotions in logical reasoning are beginning to do this (Blanchette, 2006; Goel and Vartanian, 2010). However encouraging, this is not sufficient. We need to cast a much broader net and incorporate the type of phenomena identified here. Failure to do so will result in incomplete and distorted theories of reasoning. Broadening the research program means developing experimental paradigms to study the role of instinctual biases on decision-making and using these data to inform cognitive theory. I believe that incorporating these data will point us toward something like an adulterated rationality account of reasoning.

Furthermore, cognitive psychology has emphasized the importance of top-down cognitive inhibitory processes in understanding human behavior. We know something about the neuropsychology of these processes (Shallice and Cooper, 2011). However, the adulterated rationality model, in identifying the importance of bottom-up, non-cognitive, instinctual processes, and recognizing individual differences, suggests that this focus is only half of the story. Deviation of behavior from expected norms may not simply be a function of failure of top-down control, but individual differences in the strength of the bottom-up processes. If this is the case, it would have important consequences for our legal and social norms and expectations.

Thus in summary, I am drawing attention to ubiquitous, real-world, reasoning paradigms where rational choice is modulated by instinctual biases. I argue that existing models of logical reasoning cannot adequately accommodate these phenomena and propose an adulterated rationality account of reasoning. The ubiquitousness of the phenomena call for data collection, model fitting and exploration of consequences for social and legal norms.

## Conflict of Interest Statement

The author declares that the research was conducted in the absence of any commercial or financial relationships that could be construed as a potential conflict of interest.

## Footnotes

1 The phenomenon disappears if one of the conjuncts is “Linda is active in the feminist movement.” or if the conjunction is “Linda is a bank teller and is 43 years old.”

## References

[B1] Bermudez J. L. (2002). “Rationality and psychological explanation without language,” in *Reason and Nature: Essays in the Theory of Rationality* eds Bermudez J. L. Millar A. (New York: Oxford University Press) 233–264

[B2] Berry B. (2007). *Beauty Bias: Discrimination and Social Power*. London: Greenwood Publishing Group, Inc. 10.1037/0033-295X.85.1.1

[B3] Blanchette I. (2006). The effect of emotion on interpretation and logic in a conditional reasoning task. *Mem. Cognit.* 34 1112–1125 10.3758/BF03193257 17128609

[B4] Braine M. D. S. (1978). On the relation between the natural logic of reasoning and standard logic. *Psychol. Rev.* 85 1–21 10.1037/0033-295X.85.1.1

[B5] Buss D. M. (2005). *The Handbook of Evolutionary Psychology* 1st Edn. Hoboken, NJ: Wiley

[B6] Cassirer E. (1944). *An Essay On Man: An Introduction to a Philosophy of Human Culture*. New Haven: Yale University Press

[B7] Cosmides L. (1989). The logic of social exchange: has natural selection shaped how humans reason? Studies with the Wason selection task [see comments]. *Cognition* 31 187–276 10.1016/0010-0277(89)90023-1 2743748

[B8] Cosmides L. Tooby J. (1994a). Beyond intuition and instinct blindness: toward an evolutionarily rigorous cognitive science. *Cognition* 50 41–77 10.1016/0010-0277(94)90020-5 8039372

[B9] Cosmides L. Tooby J. (1994b). “Origins of domain specificity: the evolution of functional organization,” in *Mapping the Mind: Domain Specificity in Cognition and Culture* eds Hirschfeld L. Gelman S. (NewYork: Cambridge University Press)

[B10] Cox J. R. Griggs R. A. (1982). The effects of experience on performance in Wason’s selection task. *Mem. Cognit.* 10 496–502 10.3758/BF03197653 7176911

[B11] Daly M. Wilson M. (2005). The “Cinderella effect” is no fairy tale. *Trends Cogn. Sci.* 9 507–508; author reply 508–510 10.1016/j.tics.2005.09.007 16213186

[B12] Daly M. Wilson M. I. (1994). Some differential attributes of lethal assaults on small children by stepfathers versus genetic fathers. *Ethol. Sociobiol.* 15 207–217 10.1016/0162-3095(94)90014-0

[B13] Dipboye R. L. Fromkin H. L. Wiback K. (1975). Relative importance of applicant sex, attractiveness, and scholastic standing in evaluation of job applicant resumes. *J. Appl. Psychol.* 60 39–43 10.1037/h0076352

[B14] Duchaine B. Cosmides L. Tooby J. (2001). Evolutionary psychology and the brain. *Curr. Opin. Neurobiol.* 11 225–230 10.1016/S0959-4388(00)00201-4 11301244

[B15] Evans J. (2003). In two minds: dual-process accounts of reasoning. *Trends Cogn. Sci.* 7 454–459 10.1016/j.tics.2003.08.012 14550493

[B16] Evans J. Over D. E. (1996). *Rationality and Reasoning*. New York, NY: Psychology Press

[B17] Fiddick L. Cosmides L. Tooby J. (2000). No interpretation without representation: the role of domain-specific representations and inferences in the Wason selection task. *Cognition* 77 1–79 10.1016/S0010-0277(00)00085-8 10980253

[B18] Fodor J. A. (1983). *The Modularity of Mind: An Essay on Faculty Psychology.* Cambridge, MA: MIT Press

[B19] Fodor J. A. (2001). *The Mind Doesn’t Work That Way.* Cambridge, MA: MIT Press

[B20] Gazzaniga M. S. (1998). *The Mind’s Past*. Berkeley: University of California Press

[B21] Gigerenzer G. (2007). *Gut Feelings*. New York: Viking

[B22] Gladwin T. E. Figner B. Crone E. A. Wiers R. W. (2011). Addiction, adolescence, and the integration of control and motivation. *Dev. Cogn. Neurosci.* 1 364–376 10.1016/j.dcn.2011.06.008 22436562 PMC6987561

[B23] Goel V. (2007). Anatomy of deductive reasoning. *Trends Cogn. Sci.* 11 435–441 10.1016/j.tics.2007.09.003 17913567

[B24] Goel V. (2008). “Fractionating the system of deductive reasoning,” in *The Neural Correlates of Thinking* eds Pppel E. Gulyas B. Kraft E. (New York: Springer Science)

[B25] Goel V. Dolan R. J. (2003). Explaining modulation of reasoning by belief. *Cognition* 87 B11–B22 10.1016/S0010-0277(02)00185-3 12499108

[B26] Goel V. Vartanian O. (2010). Negative emotions can attenuate the influence of beliefs on logical reasoning. *Cogn. Emot.* 25 121–131 10.1080/02699931003593942 21432659

[B27] Gregory S. Gallagher T. (2002). Spectral analysis of candidates’ nonverbal vocal communication: predicting US presidential election. *Soc. Psychol. Q.* 65 298–308 10.2307/3090125

[B28] Hosoda M. Stone-Romero E. F. Coats G. (2003). The effects of physical attractiveness on job-related outcomes: a meta-analysis of experimental studies. *Pers. Psychol.* 56 431–462 10.1111/j.1744-6570.2003.tb00157.x

[B29] Johnson-Laird P. (2006). *How We Reason*. Oxford: Oxford University Press

[B30] Kurzban R. (2012). *Why Everyone (Else) Is a Hypocrite: Evolution and the Modular Mind.* Princeton: Princeton University Press

[B31] Langlois J. H. Kalakanis L. Rubenstein A. J. Larson A. Hallam M. Smoot M. (2000). Maxims or myths of beauty? A meta-analytic and theoretical review. *Psychol. Bull.* 126 390–423 10.1037/0033-2909.126.3.390 10825783

[B32] Newell A. (1990). *Unified Theories of Cognition.* Cambridge, MA: Harvard University Press

[B33] Over D. (2002). “The rationality of evolutionary psychology,” in *Reason and Nature: Essays in the Theory of Rationality* eds Bermudez J. L. Millar A. (New York: Oxford University Press) 187–207

[B34] Pinker S. (1997). *How the Mind Works*. New York: Norton & Co 10.1111/j.1749-6632.1999.tb08538.x 10415890

[B35] Politzer G. Noveck I. A. (1991). Are conjunction rule violations the result of conversational rule violations? *J. Psycholinguist. Res.* 20 83–103 10.1007/BF01067877

[B36] Pylyshyn Z. W. (1984). *Computation and Cognition: Toward a Foundation for Cognitive Science.* Cambridge, MA: MIT Press

[B37] Rauch J. (1995). Short guys finish last. *The Economist*. Available at: http://www.jonathanrauch.com/jrauch_articles/2004/08/short_guys_fini.html (accessed December 23 1995)

[B38] Rips L. J. (1994). *The Psychology of Proof: Deductive Reasoning in Human Thinking*. Cambridge, MA: MIT Press

[B39] Shallice T. Cooper R. (2011). *The Organization of Mind.* Oxford: Oxford University Press

[B40] Sloman S. A. (1996). The empirical case for two systems of reasoning. *Psychol. Bull.* 119 3–22 10.1037/0033-2909.119.1.3

[B41] Stanovich K. (2004). *The Robot’s Rebellion: Finding Meaning in the Age of Darwin*. Chicago: University of Chicago Press. 10.7208/chicago/9780226771199.001.0001

[B42] Tversky A. Kahneman D. (1983). Extensional versus intuitive reasoning: the conjunction fallacy in probability judgment. *Psychol. Rev.* 90 293–315 10.1037/0033-295X.90.4.293 PMC4658440 26635712

[B43] Wason P. C. Shapiro D. A. (1971). Natural and contrived experience in a reasoning problem. *Q. J. Exp. Psychol.* 23 63–71 10.1080/00335557143000068

[B44] Wiers R. W. Gladwin T. E. Hofmann W. Salemink E. Ridderinkhof K. R. (2013). Cognitive bias modification and cognitive control training in addiction and related psychopathology mechanisms, clinical perspectives, and ways forward. *Clin. Psychol. Sci.* 1 192–212 10.1177/2167702612466547

